# The First Generative AI Prompt-A-Thon in Healthcare: A Novel Approach to Workforce Engagement with a Private Instance of ChatGPT

**DOI:** 10.1371/journal.pdig.0000394

**Published:** 2024-07-23

**Authors:** William R. Small, Kiran Malhotra, Vincent J. Major, Batia Wiesenfeld, Marisa Lewis, Himanshu Grover, Huming Tang, Arnab Banerjee, Michael J. Jabbour, Yindalon Aphinyanaphongs, Paul Testa, Jonathan S. Austrian

**Affiliations:** 1 Department of Health Informatics, NYU Langone Health, New York, New York, United States of America; 2 Department of Medicine, NYU Grossman School of Medicine, New York, New York, United States of America; 3 Department of Ophthalmology, NYU Grossman School of Medicine, New York, New York, United States of America; 4 Department of Population Health, NYU Grossman School of Medicine, New York, New York, United States of America; 5 Department of Management and Organizations, NYU Stern School of Business, New York, New York, United States of America; 6 Microsoft, Seattle, Washington, United States of America; 7 Department of Emergency Medicine, NYU Grossman School of Medicine, New York, New York, United States of America; University of Zurich Faculty of Medicine: Universitat Zurich Medizinische Fakultat, SWITZERLAND

## Abstract

**Background:**

Healthcare crowdsourcing events (e.g. hackathons) facilitate interdisciplinary collaboration and encourage innovation. Peer-reviewed research has not yet considered a healthcare crowdsourcing event focusing on generative artificial intelligence (GenAI), which generates text in response to detailed prompts and has vast potential for improving the efficiency of healthcare organizations. Our event, the New York University Langone Health (NYULH) Prompt-a-thon, primarily sought to inspire and build AI fluency within our diverse NYULH community, and foster collaboration and innovation. Secondarily, we sought to analyze how participants’ experience was influenced by their prior GenAI exposure and whether they received sample prompts during the workshop.

**Methods:**

Executing the event required the assembly of an expert planning committee, who recruited diverse participants, anticipated technological challenges, and prepared the event. The event was composed of didactics and workshop sessions, which educated and allowed participants to experiment with using GenAI on real healthcare data. Participants were given novel “project cards” associated with each dataset that illuminated the tasks GenAI could perform and, for a random set of teams, sample prompts to help them achieve each task (the public repository of project cards can be found at https://github.com/smallw03/NYULH-Generative-AI-Prompt-a-thon-Project-Cards). Afterwards, participants were asked to fill out a survey with 7-point Likert-style questions.

**Results:**

Our event was successful in educating and inspiring hundreds of enthusiastic in-person and virtual participants across our organization on the responsible use of GenAI in a low-cost and technologically feasible manner. All participants responded positively, on average, to each of the survey questions (e.g., confidence in their ability to use and trust GenAI). Critically, participants reported a self-perceived increase in their likelihood of using and promoting colleagues’ use of GenAI for their daily work. No significant differences were seen in the surveys of those who received sample prompts with their project task descriptions

**Conclusion:**

The first healthcare Prompt-a-thon was an overwhelming success, with minimal technological failures, positive responses from diverse participants and staff, and evidence of post-event engagement. These findings will be integral to planning future events at our institution, and to others looking to engage their workforce in utilizing GenAI.

## Introduction

Generative artificial intelligence (GenAI) use exploded with ChatGPT’s release to the public in late 2022. Large language models (LLMs), which learn from vast amounts of text data to "understand” the context in which words appear and ultimately, their meaning, belong to a class of artificial intelligence (AI) called GenAI. [1,2] ChatGPT sparked a user interface revolution in AI, making the extensive knowledge base that LLMs like GPT-3 and GPT-4 amass more accessible to everyone through natural language, rather than code, as a prompt to generate responses that mimic human syntax. [1–3] Several industries have recognized the advantages GenAI can offer, as studies with consultants and customer service agents showed increased worker productivity and reductions in employee attrition when they provided access to GenAI tools to their workforce. [4–5] For healthcare organizations to realize GenAI’s transformative potential, they must upskill their workforces in its responsible use or risk breaches of patients’ or researchers’ data privacy and perpetuation of biases that exist in training data and user prompts. [6–8]

In the spring of 2023, NYULH, a large academic medical center in the New York City area, developed and publicized workforce policies for the use of the public ChatGPT application and launched a secure and private ChatGPT-like instance as a user interface for the use of GenAI models like GPT-4. All members of the workforce were invited to apply for access to this instance and experiment with data not allowed within the public instance, such as patient information and intellectual property. Leaders from the NYULH MCIT Department of Health Informatics and the Predictive Analytics Unit helped supervise and guide this exploration. In response to the workforce’s profound interest in this technology, and the risks of them using it without proper training (e.g., putting patient health information into the public instance of ChatGPT), we needed an efficient method for engaging and educating our community that promoted collaboration and innovation. Consequently, we decided to hold an in-person event: the first GenAI “Prompt-a-thon" in healthcare, to bring together staff from all corners of our institution to learn from local experts, experiment with GenAI, and share ideas about how GenAI could revolutionize their work.

Our Prompt-a-thon builds on the burgeoning body of literature on crowdsourcing contests (e.g. hackathons, datathons, etc), which have successfully promoted innovation, utilization of new technologies, and inter-professional collaboration within healthcare communities. [3,9–19] For example, a hackathon for junior surgical physician-scientists exemplified how these events can foster innovation by accelerating academic productivity. [15] Furthermore, Aboab et al. reported that incorporating actual healthcare data into these workshops led to the development of decision support tools and novel research directly applicable to real-world tasks. [10] Others have showcased how these events equip participants with transferable skills relevant to their daily work [16], but to realize post-event success, organizers must ensure participating teams are diverse in their demographic attributes and technical capabilities. [15] Healthcare hackathons which promote GenAI utilization with real-world data [5] and involve a diverse community [15] thus have the potential to educate early adopters, upskill an entire workforce, and revolutionize patient care.

The best method of incorporating LLMs within a healthcare hackathon is unclear. We consulted various precedents for hackathon best practices, [9–12] and whether to favor determinism (e.g., highly structured workshops) versus creativity (e.g., allowing individuals maximal freedom to dictate their problem-solving approach during workshops). In favor of determinism, best practices posed by Silver et al. state that healthcare hackathons should define the purpose to stakeholders, select an apt theme or problem, choose the right time and venue, and ensure clear expectations for all participants. [9] In favor of creativity, Falk et al. underscored the significance of the participant experience and the need for event customization to encourage diverse participation. [12] To study the impact of tipping the scales towards determinism or creativity on participants’ experience with the technology and event, we decided to perform an intervention where some groups received more structure during the workshop portion of the event than others, which came in the form of curated sample prompts designed to help participants achieve various tasks with their dataset.

There were several objectives in organizing this event: i) educate the workforce on the benefits and responsible use of GenAI, ii) promote collaboration and innovation both during and after the event, and iii) examine the effects of prior GenAI exposure and the provision of sample prompts during the event on participant experience. The findings from the inaugural Prompt-a-thon will inform future approaches to engage our community in leveraging GenAI to improve healthcare.

## Methods

### Planning committee

**Fig 1:**
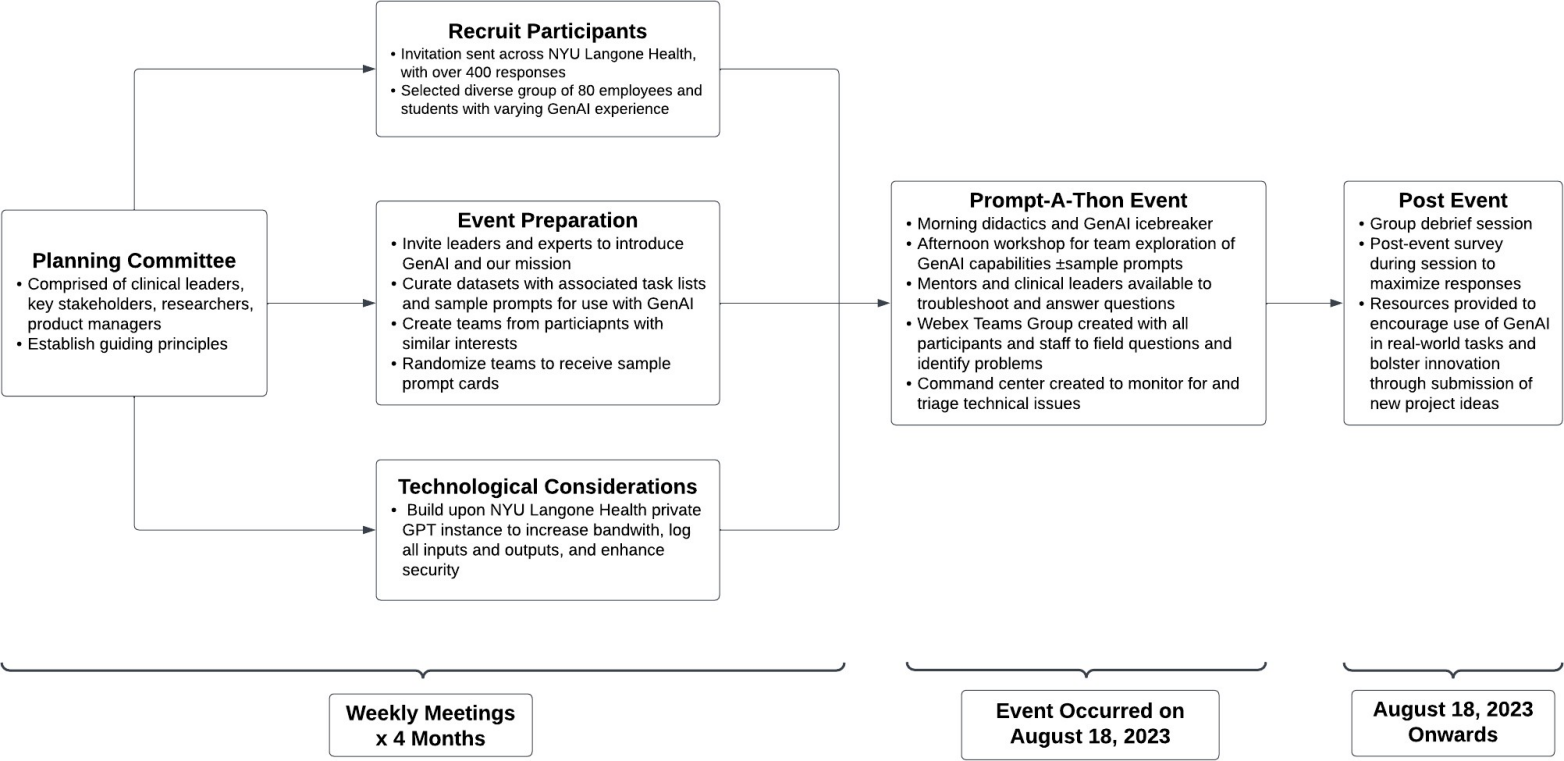
Flowchart of Event Planning and Execution.

To fulfill our objectives, we assembled a large team of clinical leaders, researchers, information technology experts, product managers, and event planners to organize and execute this event (Fig 1). Key stakeholders from the Institute in Innovation in Medical Education, office of clinical research, and the Institute of Excellence in Health Equity were also engaged in the planning and execution of the event. The Generative AI advisory board, under the leadership of the Chief Digital Information Officer and Chief Medical Information Officer, provided continual feedback to this leadership group. The planning committee agreed on the goals of the event and established a series of principles to guide its design: 1) The event would be open to all members of the workforce and all levels of skill with GenAI. 2) The event would leverage real, maximally de-identified health system data. 3) The event planning and execution would need to be both prescriptive and flexible. 4) The event must prioritize continual engagement with participants after the event. After four months of planning, the Prompt-a-thon was held on August 18, 2023 at the NYU Grossman School of Medicine.

### Recruiting participants

To engage a diverse population of the NYU community, the committee sent an email invitation to the entire NYU Langone Health workforce for the event. The questionnaire asked potential participants of their current role and department at NYU Langone, prior experience with GenAI, their primary interest in using GenAI (clinical, research, education, etc.), and any preliminary project ideas they had involving GenAI. The committee received over 400 responses, and ultimately had to select up to 80 in-person participants (due to physical space and bandwidth limitations, as well as the desire to maintain a small participant-staff ratio) to attend the workshop, while the rest were invited to attend the didactics sessions virtually. The committee sought to engage a heterogeneous group of individuals with diverse roles (physicians, nurses, social workers, music therapists, researchers), specialties, levels of influence (student vs leader), and prior experience using GenAI (no experience vs some experience vs “swear by it”) who had determination to learn about and use GenAI as evidenced in their questionnaire.

### Technological considerations

**Fig 2:**
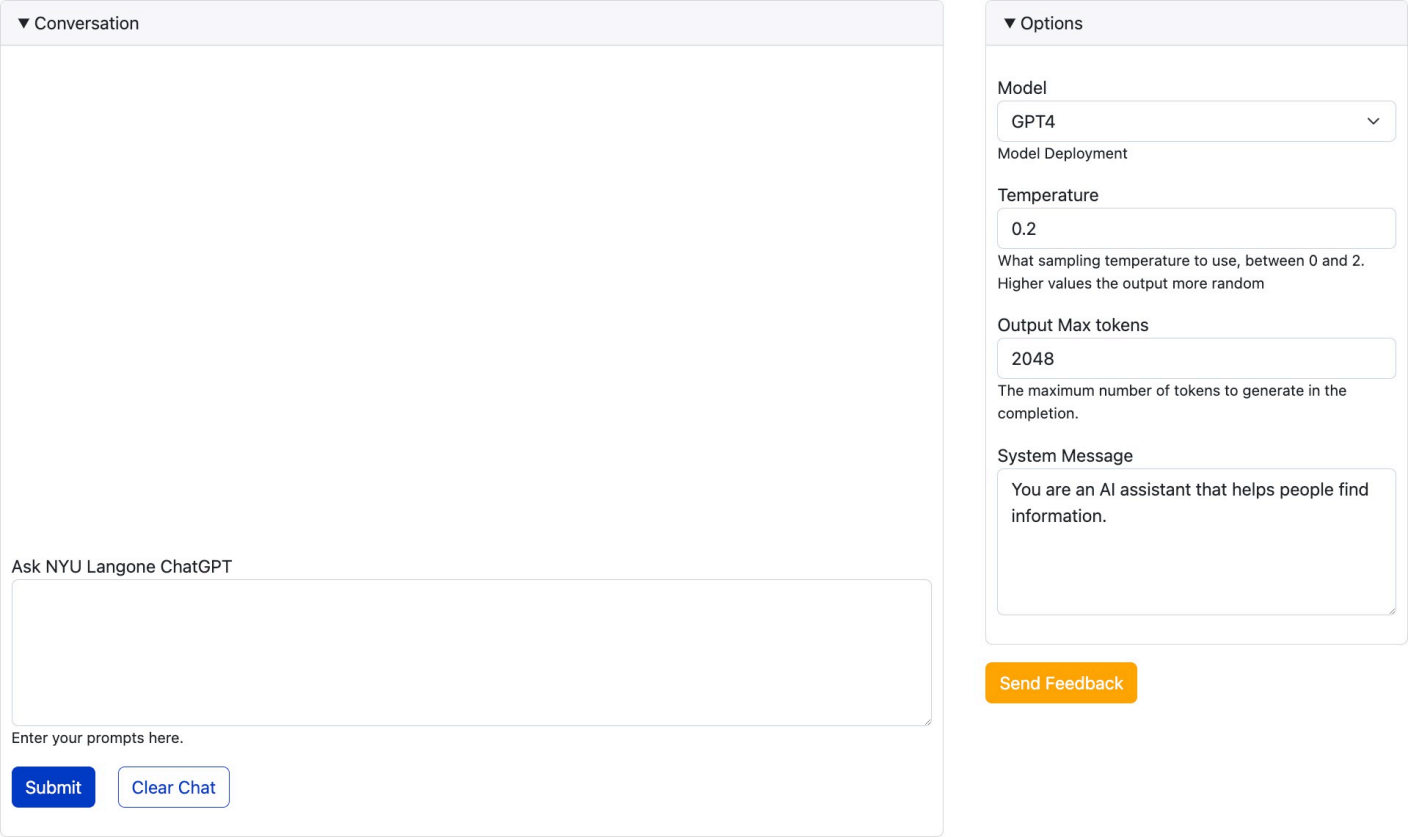
Screenshot of NYULH private instance of ChatGPT. The “Conversation” section is where users would input de-identified healthcare data along with their request, view outputs, and send follow-up messages to refine the outputs. The “Options” section allows users to choose between GenAI models (with GPT-4 as default), alter the model temperature (or degree of randomness within outputs), view the maximum output length, and change the system message (or set of instructions GenAI uses to create its responses). The “Saved Queries” tab at the top allows users to view their past inputs and outputs.

Technology was a key enabler of the Prompt-a-thon, and the components used to support the event are outlined in Table 1. All participants were granted access to NYU Langone Health’s GPT Studio (Fig 2), a playground-style user interface built on top of our private, HIPAA-compliant instances of GPT within Microsoft Azure Open AI. However, access alone was insufficient to ensure that participants would have a seamless experience in their experimentation. Bandwidth is a critical challenge with large language models; high frequency requests and long inputs and outputs saturate it and cause rate-limit errors. Our GPT instances are policed with strict limits on both requests and ‘tokens’ (the atomic unit of LLMs such as GPT). Given the expected rate of both requests and tokens used, MCIT developed both technical and educational strategies to mitigate blocked prompts.

**Table 1:** Technologies employed to support the use of GPT models for the Prompt-a-thon event and their reason for implementation.

Technologies	Function	Purpose
Microsoft Azure Open AI GPT-3.5/GPT-4	Core GPT LLM Engine	Secure GenAI instance to be used during the event
Kong API Management	API Management Tool	Facilitating API load balancing, secure access, and traffic management
Drupal 10 CMS	Content Management Platform	Used to build custom frontend and manage user access
Microsoft Azure AD	Single Sign On Authentication	Simplify sign-on to NYU Langone users
Grafana/Prometheus and Microsoft Azure GPT Resource Monitoring	Real-time Usage Monitoring Dashboard	Monitor prompt submission and bandwidth levels during event
Rancher Kubernetes Cluster	Hosting Environment	Host custom front end, API management, and monitoring dashboard
Docker Containers	Deployment Technology	Deploying custom front end stack

Technical strategies to limit mishaps included partnering with Microsoft for increased subscription resources and limiting outside workforce access to our GPT instances for all non-mission critical use for the duration of the event. Crucial to the success of the event was the development of two pieces of internal GenAI infrastructure, 1) an API (application programming interface) management system (built using a tool called Kong) and 2) the creation of a custom, branded user-interface we named NYU GPT Studio (built with a platform called Drupal) that replicates the core functionality of ChatGPT and the Azure OpenAI Studio Playground but leverages the API management system to be able to load balance between our various GPT deployments (e.g. GPT-8k and GPT-32k instances) and monitor their utilization. NYU GPT Studio provided additional user-experience benefits such as offering participants the ability to log and retrieve every query and response for future reference in a tab called “Saved Queries.” For the Prompt-a-thon, we designed a simple, user-friendly interface that included direct links to the data sets, projects, and our GenAI help email. The front end also provided enhanced security due to single sign-on for NYU Langone users.

Another set of strategies to prevent errors were educational in nature. This included explanation of the various (and complex) limitations of GPT models (content window, max output length, request and token per minute limits). Expectations were thus set for participants that these errors might occur, and facilitated education on how to troubleshoot these errors, which included rules of thumb such as wait 10 seconds after an error before re-attempting, use a model with more capacity (i.e. GPT-3.5 vs GPT-4) when having consistent issues, minimize input size (i.e., including one section of a document rather than the entire thing), and clear the chat between different attempts or reduce the number of prior messages in a conversation that GPT takes into account to prevent a conversation hitting the context limit. For the purposes of the Prompt-a-thon, we defaulted NYU GPT Studio to use GPT-3.5 during the first half of the event when surge demand was expected, and we curated all provided data sources to be less than 4,000 tokens to keep within token limits.

In anticipation of technological issues throughout the event, we maintained a high ratio of mentors to participants and established a command center, in which technological experts monitored the event via a real-time dashboard displaying usage volumes and errors. Multiple group chatrooms were created and monitored by event staff on the collaborative platform, Webex Teams, to allow participants to ask questions or provide feedback throughout the day, and internally, for staff to troubleshoot technological difficulties. Our contingency plan in case of an Azure OpenAI outage was to instruct participants to create an account with the public ChatGPT website (chat.openai.com) and have the command center limit shared data sources to only those that were publicly available.

### The Prompt-A-Thon Event

We emulated the format of several peer-reviewed crowdsourcing events. [3,9–12] Our two-pronged event included both a didactics session, which utilized experts across NYULH to educate participants about how to use GenAI, ethical considerations when using it, and the potential of LLMs more generally, and an interactive workshop in which participants could engage with a private instance of GPT models using real-world clinical and research data.

Selected participants were invited to join in-person, while the rest were invited to attend the didactic sessions virtually on the day of the event. Event staff wore violet shirts with the theme “Keep Calm and Prompt-a-Thon,” so that participants could identify them.

### Morning didactics session

After breakfast, participants were gathered into a conference room where they heard several 20-minute lectures from clinical, research, and ethics leadership in GenAI, which taught participants basic techniques for using GenAI, highlighted potential applications of GenAI, and underscored the necessity for participants to critically evaluate the output of their prompts for accuracy and potential biases. After introductory lectures, participants had their first experience using GenAI with an icebreaker “sniglet” challenge: prompt GenAI to create a novel word and definition related to healthcare. The audience then voted on the best word using Slido [20], a real time polling website, which was instrumental in energizing and priming them for more advanced topics. The lecture, “Ethical Considerations of Generative Artificial Intelligence in Healthcare,” highlighted a case study that displayed the potential of AI to perpetuate health inequalities by exposing a significant racial bias in a widely used health system algorithm, [8] leading to the recommendation that users should remain vigilant for such issues during development of GenAI applications in healthcare and seek feedback from a diverse audience. To address data privacy concerns around using public LLMs like ChatGPT, internal GenAI policy guidelines were reviewed permitting NYULH employees to input sensitive data types (e.g., patient health information, clinical or human subjects research data, or intellectual property such as grant proposals) into GenAI only when using a private instance of GPT like Azure Studio.

The inherent technological limitations of GenAI were addressed multiple times, including its lack of common sense and the potential to perpetuate bias that exists in its training data or prompts. These limitations can manifest in various ways, such as hallucinations (fabricated outputs not based on evidence), omissions (failure to include important or requested information), inaccurate inferences (incorrect conclusions drawn from accurate and relevant data), and sycophancy (outputs compromising accuracy in the service of being responsive to user expectations conveyed in prompts). [21–25] Participants were introduced to techniques for reducing hallucination, such as providing explicit instructions, constraining answers to a source database or a pre-specified format, and having the model acknowledge its own limitations (instruct it to say “I don’t know” if it doesn’t have enough information to answer the question). Experimentation with advanced methods like “chain of thought” prompting, [21] which extracts GenAI’s “reasoning” for its output, was encouraged. This technique facilitates human review of inappropriate associations and assumptions that can lead to faulty outputs and allows users to address them in subsequent prompts. [21–22] Participants thus spent the morning learning how to use GenAI, understanding its limitations, and discovering strategies to overcome them, while, most importantly, becoming aware of their responsibility to independently verify any GenAI content they plan to utilize in their professional work.

### Afternoon workshop session

For the interactive workshop portion of the event, we divided the participants into twenty clinically- or research-focused teams of four based on their self-defined interests (research, clinical, or educational) from the pre-event questionnaire. We sought to maintain high similarity in the interests of team’s participants while maximizing diversity in prior experience with GenAI and institutional role.

**Fig 3:**
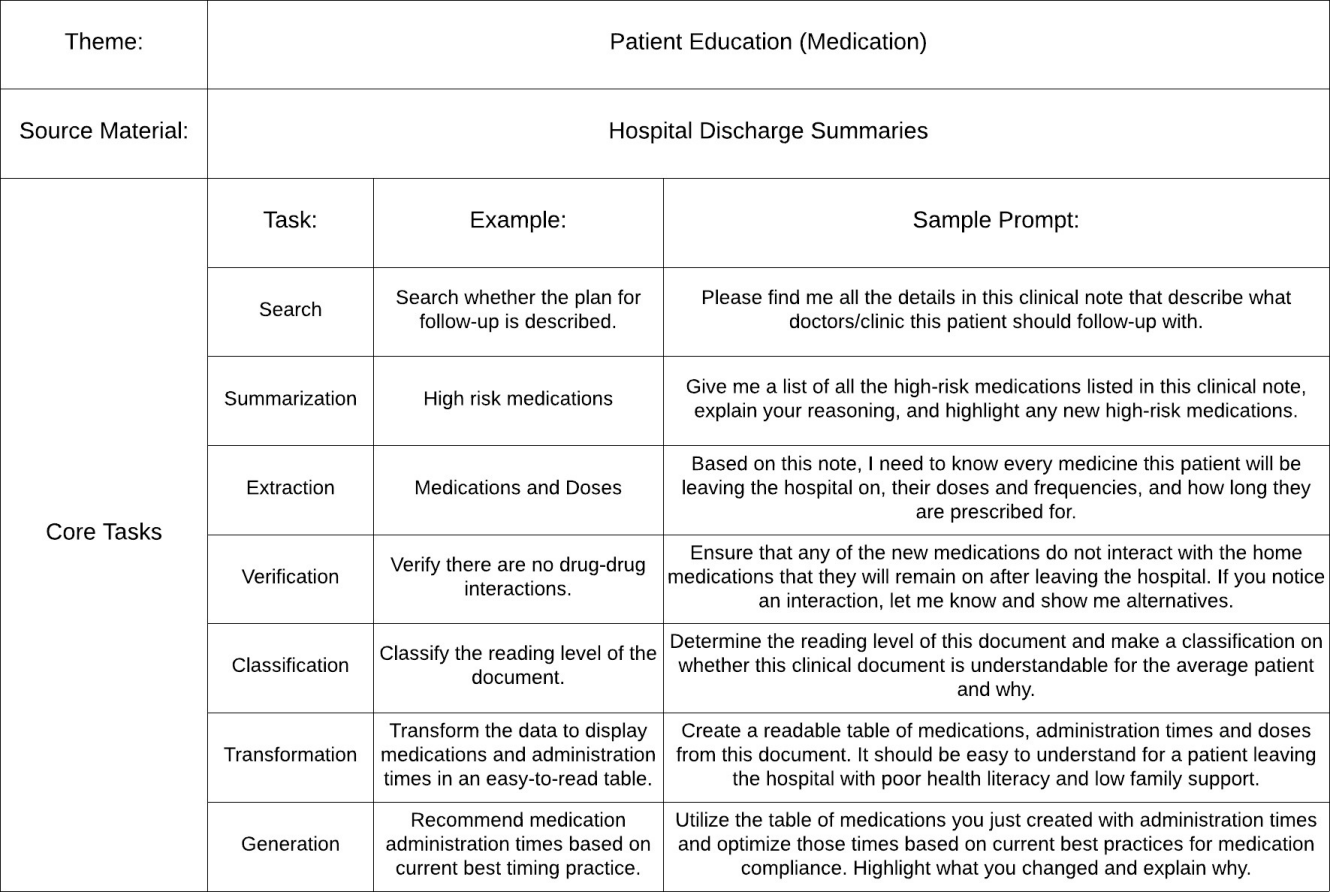
Example project card that asks participants to use GenAI on hospital discharge summaries to perform tasks related to patient education.

Potential projects that the teams could choose from were delineated by virtual “project cards” given to them beforehand (Fig 3; S1 Appendix), and comprised of an overall theme, relevant data sources, and what we described as core tasks (e.g. summarization or transformation of a text). Themes were derived directly from the participant responses and supplemented by committee members, resulting in 15 clinical teams with themes of patient education, diagnosis/treatment, and equity, and 5 research teams focused on grant proposal and literature summarization. Datasets were composed of de-identified real-world clinical notes, patient-provider message-response pairs, flowsheet templates, actual grant applications, published articles, and medical education goals. All datasets were maximally de-identified through manual review by at least two independent reviewers who obscured all sensitive patient information with placeholders (e.g. names replaced with “[patient name]” or “[provider name]”).

The project cards aimed to make prompt engineering less intimidating for our novice users. To create them required taxonomizing GenAI’s capabilities into distinct categories inspired by the literature on prompt engineering. [21–25] We found existing prompt engineering frameworks too broad and complex for our use-cases, [24–25] and wanted to provide participants with simple tasks that could be transferable to their work. The seven distinct categories included search, summarization, extraction, verification, classification, transformation, and generation. We provided contextualized examples of how each task could be applied to data sources. For instance, users were explicitly tasked with transforming clinical notes into patient-friendly versions using simpler language.

Over a working lunch, participants met their team members and mentors, and discussed their initial approach to choosing and executing on a project related to their assigned theme. Once they arrived at their assigned rooms for the afternoon workshop, teams had two hours to explore the capabilities of GenAI within our NYU GPT Studio and complete a small number of core tasks. Individuals were given the freedom to iterate upon the existing projects or use the available data sources for novel projects related to their work.

A mentor from the Department of Health Informatics data science team was assigned to facilitate up to two teams. Furthermore, physician and nurse informaticists staffed each room. Mentors evaluated their teams on the quality of their collaboration, growth during the workshop, prompt outputs, and ultimately, the suitability of their top project for implementation into our health system.

### Event Wrap-Up

After the workshop, the participants attended an awards ceremony, and were informed about how they could continue their GenAI journey with continued access to NYU GPT Studio, GenAI office hours, and a moderated group WebEx chat. They were encouraged to submit any future project ideas to our Department of Health Informatics for possible widescale implementation.

Awards were given based on the mentors’ evaluations. The top overall award, or “NYULH GenAI Visionaries Award”, was given to a research-oriented team who effectively generated specific aims from summaries of various research projects. Other winners worked on patient friendly explanations of lab results, patient friendly hospital discharge summaries, and automatic review of grant proposals’ research methods for bias related to health equity.

### Evaluation

Participants completed an online survey (Qualtrics) (Table 2) before the awards ceremony, so as not to bias their answers based on whether they won an award. Response scales for all items assessed agreement from 1 (not at all) to 7 (very strong). The survey also had a free text section to elicit general feedback.

**Table 2:** List of survey questions answered by respondents towards the end of the event.

Survey Questions (7-point Likert)
Today’s Prompt-a-thon increased my ability to engineer prompts to get the desired output.
Today’s Prompt-a-thon increased how efficiently I will be able to perform job tasks using generative AI.
Today’s Prompt-a-thon increased my tendency to trust outputs from generative AI.
Today’s Prompt-a-thon increased my confidence performing various tasks within generative AI.
Today’s Prompt-a-thon increased my comfort level using generative AI.
Today’s Prompt-a-thon increased the likelihood that I will propose a healthcare related generative AI project.
Today’s Prompt-a-thon increased the likelihood that I will encourage my colleagues to attend a Prompt-a-thon.
My prompts during the event were creative.
My prompts during the event were effective at accomplishing the task.

Feedback from the mentors was gathered in a debrief focus group session, where they were asked to elucidate what went well or poorly from their perspective, and describe how teams progressed throughout the workshop session, including the questions participants asked and how well they worked together.

After the event, we continued to engage with participants via the shared Webex Teams group chat to answer their questions about GPT and share resources. One of the key measures of engagement was requests for access to the NYU GPT Studio tool.

### Analyses

Along with calculating the average score for each survey response, we conducted one-way ANOVAs to evaluate the effects of both prior GenAI experience and the provision of sample prompts to participants on their perceptions of the Prompt-a-thon’s impact on them. Our study’s design and the event’s findings were completed in accordance with the STROBE guidelines for observational studies (see S2 Appendix). [26]

### Ethics statement

This study met NYU criteria for quality improvement work and did not undergo IRB review. Participants were not required to complete the survey, and upon scanning the QR code during the wrap-up session, provided verbal consent that their data would be utilized for quality improvement purposes.

## Results

### Participants

There were 412 applicants for our event, and the committee selected 90 participants. There were 70 attendees and 56 staff members on the day of the event, and an additional 375 virtual attendees of the didactic sessions.

The group of attendees was diverse, including 38 females (54.3%) and 32 males (45.7%). Health care professionals made up 47.1% of the total participants (n = 33), while 14.3% consisted of researchers (n = 10), and 38.6% administrative roles (n = 27). Of the 61 survey responders, 67.2% reported prior experience with GenAI (n = 41), with 8.2% (n = 5) having extensive experience (“swore by it”), 16.4% never used GenAI (n = 10), and 8.2% did not answer the question (n = 5)

### Event Metrics and Post-Event engagement

There were 1944 prompts submitted from 9AM-4PM, predominantly concentrated within the two hours of our workshop portion (12:30PM–2:30PM) where 1310 prompts (67.4%) were submitted. The mean rate of prompt submission was 10.9 prompts/minute with a max of 26 prompts/minute, median [IQR] of 10.5 [7–15] per minute. These rates were substantially below our imposed requests per minute limit of 360 for GPT-4 8k.

There were only four blocked events where a group of around fifteen users ran prompts almost simultaneously, surpassing the tokens per minute rate-limit. During the morning, 62% of the prompts used GPT-3.5 (compared to 10% after 12PM) as this was defaulted in NYU GPT Studio to handle the expected high demand during the ice-breaker session. We observed a period of thirteen consecutive minutes where there was a sustained demand greater than fifteen requests per minute with a peak of fifty-three. All these rates were below our resource rate-limits, so we could have handled the demand with GPT-4 alone.

Due to a storage formatting issue, the text of 51.2% (995 out of 1944) of the prompts were unavailable for secondary analysis. From the remaining 949 prompts, mean token length was 1830 input tokens (median [IQR]: 950 [283, 2348]) and 274 output tokens (median [IQR]: 241 [127, 370]). The average (and median) number of prompts per user was 24, with a range from 1 to 74 prompts/user. For the entire day, across all GPT resources, our total spend on GPT was less than $300.

During the week including our Prompt-a-thon, we received 104 access requests compared to a weekly median of 15 (IQR [10–17]) before and 15 (IQR [6–22]) after the event. Workshop participants also continued to utilize the tool; 35% (n = 25) continued to interact with the custom front end, creating over 450 prompts in the four weeks after the event.

### Survey results

87.1% of in-person attendees (n = 61) answered the survey, and mean survey responses are below (Table 3). Participants perceived that the event most strongly improved the likelihood they would encourage a colleague to attend a Prompt-a-thon (mean = 6.7) and use GenAI in their daily work (mean = 6.3), and improved their ability to engineer prompts (mean = 6.3). Although the mean score was lower than the others, the event improved participants’ trust in GenAI (mean = 4.8).

**Table 3:** Mean scores from each of the survey questions.

Participant Survey Responses (N = 61)	
Statement	Mean Score (7-point Likert Scale)
Increased ability to engineer prompts to get the desired output	6.3
Increased efficiency performing job tasks using GenAI	5.9
Increased trust in outputs from GenAI	4.8
Increased confidence using GenAI to perform tasks	5.6
Increased comfort using GenAI	5.9
Increased likelihood of proposing a healthcare-related GenAI project	6.0
Increased likelihood of encouraging colleagues to use GenAI for healthcare	6.3
Increased likelihood of encouraging colleagues to attend a Prompt-a-thon	6.7
I felt that my prompts were creative	5.6
I felt that my prompts were effective	5.9

A participant was said to “agree” with a survey question if their response was higher than the neutral midpoint of the 7-point Likert scale. Nearly all participants (95.1%; n = 58) agreed that their ability to engineer prompts was improved by the event, while 88.5% (n = 54) reported their efficiency in using GenAI improved. Only 57.4% (n = 35) reported the event improved their trust in GenAI, though 88.5% (n = 54) reported improved confidence in performing various tasks with GenAI and 85.2% (n = 52) were more comfortable using GenAI because of the event. Large majorities of participants reported that the event increased the likelihood that they would propose a GenAI project for healthcare (85.2%; n = 52) and that they would encourage others to use GenAI for healthcare (93.4%; n = 57). Likewise, nearly all participants (96.7%; n = 59) agreed that they would encourage their colleagues to attend a Prompt-a-thon. Finally, 78.7% (n = 48) of participants thought their prompts were creative during the event and 90.2% (n = 55) believed their prompts were effective at accomplishing their desired task.

One-way ANOVAs indicate that participants’ perceptions of the Prompt-a-thon did not differ as a function of whether (or not) they were given sample prompts, suggesting that providing sample prompts did not play a meaningful role in participants’ experience of the event.

Participant’s perceptions of several aspects of the Prompt-a-thon differed as a function of their prior experience with GenAI, however (Table 4). For example, their confidence in performing tasks using generative AI increased with greater prior experience. On the other hand, those with high prior experience had lower average trust in generative AI outputs. This is possibly due to greater exposure to GenAI errors and hallucinations. Perceptions of prompt creativity were highest for those with moderate experience.

**Table 4:** Mean responses to survey questions stratified by level of prior experience with GenAI.

Survey Question After Prompt-a-thon	Prior Experience Using GenAI	Mean Score (1.0–7.0)
Increased ability to engineer prompts to get the desired output	Never Used it (N = 10)	6.10
	Tried it (N = 41)	6.41
	Swear by It (N = 5)	6.00
Increased efficiency to perform job tasks using generative AI	Never Used it	5.50
	Tried it	5.95
	Swear by It	6.00
Increased trust in outputs from generative AI	Never Used it	4.80
	Tried it	4.93
	Swear by It	3.60
Increased confidence performing tasks using generative AI	Never Used it	4.90
	Tried it	5.76
	Swear by It	6.20
Increased comfort using generative AI	Never Used it	5.20
	Tried it	6.05
	Swear by It	6.20
Increased likelihood of proposing a healthcare- related generative AI project	Never Used it	6.50
	Tried it	5.90
	Swear by It	5.60
Increased likelihood of encouraging colleagues to use generative AI for healthcare	Never Used it	6.40
	Tried it	6.32
	Swear by It	6.40
Increased likelihood of encouraging colleagues to attend a Prompt-a-thon	Never Used it	6.80
	Tried it	6.68
	Swear by It	6.20
Prompt creativity	Never Used it	4.90
	Tried it	5.88
	Swear by It	5.20
Prompt effectiveness	Never Used it	5.70
	Tried it	6.12
	Swear by It	5.60

### Comments from participants

Overall, the workshop received positive comments from participants, examples of which are in Table 5. Most attendees appreciated the event’s informative nature, dynamic discussions, and the diversity of ideas from participants, feeling inspired to undertake potential projects in their departments.

**Table 5:** Example comments left by participants within the survey, along with their role at NYULH and prior experience with GenAI.

*“This was a very informative and dynamic session*. *I learned a lot and was able to talk to a group of people with different ideas*. *The session also gave me ideas of projects that I would like to see if I can do in my department*.*”*—Dentist (Tried It)
*“Such a fantastic opportunity to participate in this*. *The expert resources were incredibly helpful*, *the access to- and participation of leadership was also tremendous*. *Very well done*. *For future promptathons it would be great to work with a more traditional data set*.*”*—Administrator (Tried It)
*“Is the question about trusting outputs a trick question*? *Trust but verify*! *Thank you for hosting this—I feel energized and I have so many wonderful thoughts and ideas running through my head*. *Today was brilliant*.*”*—Technologist (Tried It)
*“The session was very well conceived*. *The opening talks were concrete and made AI accessible*. *The example prompts from real life were really helpful*. *It was about 30 min too long*. *Thank you for hosting*.*”*—Medical Director (Tried It)
*“’I would prefer having more colleagues that I work with attending the same training to discuss how to incorporate AI into our work at different levels*.*”*—Care Coordinator (No Experience)
*”’I would have appreciated more instruction on the specifics of prompt engineering and it would have been useful to have a mentor sit with us the entire time and give more specifics about the goals of the team time*.*”*—Care Coordinator (Tried It)

Some attendees wished for additional specific instruction on prompt engineering, more detailed directions for the workshop tasks, and dedicated mentors for each team. Some reported disruptions due to noise from other surrounding working groups in the larger workrooms. Suggestions were made for follow-up Prompt-a-thon events to be more specialized, so that teams could have more people from the same department or specialty ideating how to use GenAI to improve their daily work.

### Observations from mentors

Feedback from mentors largely paralleled the sentiments of the participants. Mentors observed that participants entered the workshop portion of the event enthusiastic due to the morning presentations. However, they noticed that some were unclear about the workshop’s objectives and others chose to explore their own interests with GenAI rather than work with their team on the designated task.

Mentors predominantly offered guidance on refining prompts, dealing with the nuances of the technology (i.e., the differences between system and user prompts), and managing data input (i.e., strategies for dealing with token limits). They identified that the most successful participants were those who exhibited enthusiasm to experiment with GenAI, submitted follow-up prompts in a conversation-like fashion (rather than continually re-engineer the initial prompt), and thought deeply about how GenAI could help them in their daily work.

The lack of example prompts for some teams led to “blank page anxiety,” causing some to struggle with how to begin their exploration. However, those with access to sample prompts were observed to first follow them closely before using the results to refine their inputs.

The mentors also provided suggestions for future workshops. Those included allowing participants to work on tasks relating to their job roles and implementing “share-out” portions of the workshop where teams could learn from what the others were doing.

## Discussion

The NYULH Prompt-a-thon aimed to augment the understanding and utilization of GenAI within our workforce, with the broader goal of democratizing the technology throughout our health system. Our event was designed to demonstrate the capabilities of GenAI, encourage its use for research and clinical tasks within NYU Langone Health, and ascertain how to run an engaging and educational Prompt-a-thon for a professional, diverse healthcare community. We confirmed our hypotheses that the event would bolster participants’ confidence and comfort using GenAI while underscoring its limitations and ethical considerations.

The feasibility of future Prompt-a-thons is supported by the broad engagement of our healthcare community, our ability to effectively support the technological challenges inherent in such an event, and the low cost of technology usage during the event. The 412 and 70 virtual and in person attendees, with minimal advertising, reflect the vast interest in this type of event and its success in activating the workforce around GenAI, particularly the 18.5% percent who applied and hadn’t used GPT yet. The heterogeneity of employees who attended this event highlights the ability for such an event to reach all corners of a healthcare system. Further supporting the feasibility of this event, technological challenges such as bandwidth and privacy concerns were anticipated and prepared for, such that very few blocked calls to the API occurred and users were satisfied with their experience. Other organizations can reference our event metrics to ensure they can rightsize their technology capacity to the participant demands we observed (e.g. 24 prompts per user per session; max rates of 26 prompts per minute in a 70-attendee event).

The survey findings suggest our event was received well by most attendees, was successful in increasing participants’ confidence and comfort using GenAI, and would likely drive engagement in the broader community due to participants’ strong desire to encourage others to use GenAI and attend similar events. These results support the notion that crowd events like hackathons can foster positive participant attitudes and accelerate innovation. [9] Callcut et al. discussed how these events can foster networking with domain experts and accelerate academic productivity, [15] which aligns with the enthusiasm demonstrated by participants after the lightning talks by experts in the field and their enhanced willingness to disseminate excitement about and utilization of GenAI across the health system.

While users on average found their trust in GenAI improved because of the event, the fact that it scored the lowest of all survey questions suggests we were successful in our objective of promoting responsible use of GenAI by encouraging skepticism of GenAI outputs among participants without hampering their excitement for exploring its innovative capabilities. Recognizing the need for healthcare professionals and researchers to adapt to rapidly evolving technology, we equipped our participants with knowledge on responsible GenAI use by highlighting its potential to potentiate bias,^6–8^ data privacy issues, [4–5] and inherent technological limitations. [21–25] Crucially, we emphasized that mitigating these concerns requires the vigilance of subject-matter experts who are aware of these issues. The Prompt-a-thon’s achievement in promoting healthy skepticism of participants towards GenAI stems from their understanding of its capabilities and limitations. However, the event also instilled that participant-generated prompts could also lead to biased outputs, so they should employ prompt engineering techniques to limit or understand biases present in their prompts (e.g., chain-of-thought prompting [21–22]) and recruit diverse feedback when addressing unacceptable outputs.

The Prompt-a-thon received positive qualitative feedback from participants and mentors, who found the event informative, inspiring, and diverse, though they identified a few areas for improvement. Both groups agreed that there could have been more focus on objectives prior to beginning the workshop portion of the event, which appeared to be exacerbated in teams without sample prompts. Teams were intentionally heterogenous with respect to role, department and specialty because diverse teams have been shown to be more innovative, [19] which was a central objective for event organizers. However, mentors reported that some individuals used GenAI to address their own work-related tasks rather than collaborating with their teams. Participants’ goals likely included developing local solutions for tasks specific to their individual job role. Therefore, they may have been more motivated to collaborate if groups were more homogenous with respect to their department or specialty, which must be balanced against event organizers’ innovation-oriented objectives. To promote greater team collaboration, we could have utilized the strategy of Hynes et al., who provided participants with detailed instructions and examples weeks in advance of the event. [18] Additionally, devoting more of the didactics sessions to preparing participants for the workshop, making the workshop more structured with explicit instructions at multiple time periods, and enabling report-outs of best practices teams identified (followed by time for teams to experiment with implementing these best practices) may have further facilitated collaboration. These conclusions align with prior work stating that it is critical to set clear expectations for hackathon participants [11,18] and account for the differing goals of participants and organizers. [12]

Our experience with the Prompt-a-thon provided valuable insights into the nuances of prompt engineering for novice users in healthcare or research roles. The use of project cards, which delineated potential projects into themes, relevant data sources, and core tasks, successfully demystified the complexities of GenAI for our participants. The core tasks were inspired by existing literature on prompt engineering, [21–25] but sought to offer a simpler, more accessible framework that illuminated GenAI’s broad applicability to participants’ daily work. Providing sample prompts to a random selection of clinical teams, while having no significant impact on survey results, was found during qualitative feedback sessions with mentors to alleviate participants’ “blank page anxiety,” facilitating more rapid progress. Our experience revealed the importance of providing a structured, simplified framework to novice prompt engineers; offering distinct, well-defined tasks and examples via the project cards facilitated a smoother and more fruitful interaction with GenAI, enhancing their overall experience and learning outcomes.

The evaluation of the Prompt-a-thon has several limitations. First, we did not use validated scales because there is limited research on optimal evaluations of crowdsourcing events, especially those that involve LLMs. Our assessment of prior experience with GenAI was not sensitive enough to capture the likely underlying variance. Most participants characterized their prior GenAI experience as “moderate,” limiting our confidence in comparisons across groups stratified by experience. Second, the generalizability of the findings may be limited to those who work at large academic medical centers whose workforce may be more experienced with and supported in using new technologies. Third, our sample size was limited by event constraints, which included physical space, bandwidth, and other associated costs.

## Conclusion

In conclusion, the Prompt-a-thon successfully brought together diverse healthcare professionals to explore and engage with GenAI, demonstrating its transformative potential and accessibility. The event effectively taught participants responsible GenAI use by highlighting its limitations and associated prompt engineering solutions and fostering personal accountability for GenAI outputs employed for their professional work. It also provided valuable insights for future initiatives, emphasizing the need for more specific instruction, particularly on prompt engineering, and structure, such as providing time for participants to share effective practices as a group and then apply them. As part of our ongoing commitment to fostering a supportive GenAI community, we will continue to provide resources for participants, which includes offering support office hours. We also have “mini-Prompt-a-thons" planned with domain-specific champions and datasets to enhance engagement and provide more targeted learning experiences.

We believe the “Prompt-a-thon” intervention and associated learnings are readily scalable to other healthcare institutions interested in democratizing Gen AI. Educating our collective healthcare workforce in Gen AI’s strengths and limitations is essential to realizing the dream of this technology in improving patient care.

## Supporting information

S1 Appendix Other Project Cards Given to Participants for Guidance During the Workshop in Addition to Project Card 1 (Fig 3). A public repository of project cards can be found at https://github.com/smallw03/NYULH-Generative-AI-Prompt-a-thon-Project-Cards. (c) 2024 NYU Langone Health. All rights reserved. For commercial use please contact TOV at https://tov.med.nyu.edu.
(DOCX)

S2 Appendix STROBE Checklist for Cross-Sectional Studies [26]. (DOCX)

S1 Data Survey Data from Participants of the Prompt-a-thon. (CSV)
