# Supplementary material for: The First Generative AI Prompt-A-Thon in Healthcare: A Novel Approach to Workforce Engagement with a Private Instance of ChatGPT
Source: PLOS Digit Health. 2024 Jul 23;3(7):e0000394. doi: 10.1371/journal.pdig.0000394 (PMC11265701; doi:10.1371/journal.pdig.0000394)
Supplement: S1 Appendix — A public repository of project cards can be found at https://github.com/smallw03/NYULH-Generative-AI-Prompt-a-thon-Project-Cards. (c) 2024 NYU Langone Health. All rights reserved. For commercial use please contact TOV at https://tov.med.nyu.edu. (DOCX) [file pdig.0000394.s001.docx]

S1 Appendix – Other Project Cards Given to Participants for Guidance During the Workshop in Addition to Project Card 1 (Figure 3). A public repository of project cards can be found at <https://github.com/smallw03/NYULH-Generative-AI-Prompt-a-thon-Project-Cards>. (c) 2024 NYU Langone Health. All rights reserved. For commercial use please contact TOV at <https://tov.med.nyu.edu>

Example Project Card: Health Equity

| Theme: | Equity (Evaluate clinical data for bias) | | |
| --- | --- | --- | --- |
| Source Material: | History and Physical Notes | | |
| Core Tasks | Task: | Example: | Sample Prompt: |
|  | Search | Look for indicators of socioeconomic status: Identify indicators of the patient’s race, ethnicity, wealth, income, occupation, and/or education level. | Isolate into a list all indicators of the patient’s socioeconomic status from this clinical note. Comment on what you think their status is based on the evidence. |
|  | Summarization | Describe why the patient was admitted to the hospital. | Summarize the following clinical document and not just describe the patient’s primary problem, but whether you think any social determinants of health could have played a major role. |
|  | Extraction | Review the H&P and please extract any positive descriptors of the patient or their behavior, for example, compliments or shows of approval. | Extract any indicators of bias.  Extract any negative descriptors of the patient or their behavior, for example, resistant, refusing, agitated, difficult or noncompliant. |
|  | Verification | Ensure there is shared decision making with the patient in formulating the assessment and plan. | Please verify whether there is evidence the clinician participated with the patient in shared decision making when constructing their plan. Explain why you think so. |
|  | Classification | Rate how patient-centered the document is. | Considering the use of negative and positive descriptors and inclusion or exclusion of evidence for shared decision making, I need you to classify the H&P as high, moderate, or low in terms of patient-centered-ness. |
|  | Transformation | Increase patient trust in the medical team. | Without medical jargon, please use the H&P to create a one paragraph explanation to the patient about why they are being admitted to the hospital that communicates the team listened to their complaints and their concern for the patient’s well-being. |
|  | Generation | Create an alternative assessment and plan. | Review the ED Data and History section of the H&P to create an assessment and plan. Describe the differences between your assessment and plan and the one in the H&P. |

Example Project Card: Clinician Efficiency

| Theme: | Clinician Efficiency (Case Management) | | |
| --- | --- | --- | --- |
| Source Material: | H&P | | |
| Core Tasks | Task: | Example: |  |
|  | Search | Abnormal physical exam findings | Please list out all the abnormal exam findings and what you think that might mean in the context of this clinical note. |
|  | Summarization | Plan of Care | I am a medical student, please summarize the plan of care in this note and describe to me what you think the author’s reasoning was for each decision. |
|  | Extraction | Chronic diagnoses and correlating medications | I want you to find me all the chronic comorbidities written about in this note, and to place all the corresponding medications to each diagnosis. Please make suggestions about new medicines this patient should be on. |
|  | Verification | Diagnosis, ICD/CPT code are correct | In your opinion was the primary diagnosis or differential correct/complete? What else are you thinking about? |
|  | Classification | The condition for which they were admitted to the hospital and the projected healing/care time | Why was this patient admitted to the hospital? Determine whether you think the patient has already recovered, needs a few more days of recovery, a few more months. |
|  | Transformation | Transform data into a Case management plan of care/note | Turn this clinical note into a detailed care management plan that a social worker can go over with this patient prior to discharge. |
|  | Generation | Recommended additional modalities/care plans to aid in the timely discharge of the patient | Identify all the missing components of this note, provide recommendation on what to include in each section. Focus on ensuring timely discharge of the patient and explain how each component does that. |

Example Project Card: Diagnosis and Treatment

| Theme: | Diagnosis and Treatment | | |
| --- | --- | --- | --- |
| Source Material: | Emergency Department Notes | | |
| Core Tasks | Task: | Example: | Sample Prompt: |
|  | Search | What clinician wrote the document | Identify the writer of this document and their opinion about the situation. |
|  | Summarization | pertinent findings from the history and physical exam | Give me a paragraph that describes the patient’s physical exam in this note. Based on the rest of the note, is there anything you wish was included that is not present? |
|  | Extraction | Abnormal labs from the chart | What are all the abnormal labs present in this note? What do they mean for this patient? |
|  | Verification | Verify there are no internal inconsistencies in the document | Ensure that the writer of this document is not contradicting themselves within it. If so, identify the contradiction and what you think is correct. |
|  | Classification | Whether the diagnosis and treatment plan require the patient to stay in the hospital | Decide from this note about whether the patient is ready for discharge. Explain your reasoning. |
|  | Transformation | Transform document into summary of care for a referring provider | I am the primary care doctor who referred this patient to the service of the provider who wrote this note. Please turn this note into a summary for someone like me. |
|  | Generation | Create doctor’s note for a patient from clinical document. | Create a doctor’s note for this patient based on the below H&P that states they have an appropriate excuse for missing work and a general reason (do not state their primary diagnosis or any patient health information). |

Example Project Card: Facilitated Reading of Abstracts

| Theme: | Research: Literature and Abstracts | | |
| --- | --- | --- | --- |
| Source Material: | Abstracts of Heart Failure Review Papers.docx | | |
| Core Tasks | Task: | Example: | Sample Prompt: |
|  | Summarization | Summarize the main findings of the reviews | What are the main findings from the following papers? If there is any overlap describe if the papers agree or disagree with one another. |
|  | Extraction | Number of participants | How many study participants are in each study described? Could you list all those with similar study design in groups with that number next to them? |
|  | Classification | Classify the type of review | What type of review paper is this? Does the methodology make sense for a review of this type? |
|  | Transformation | Create a table of the results | Create an easily digestible table from the results of this paper. Act like I am a student trying to better understand research methodology. |
|  | Generation | Create a follow up research plan based on these papers | Produce a few possible studies that should follow up this paper. Describe the methodology and purpose of each. |
